# Pharmacokinetics of novel erythropoiesis stimulating protein (NESP) in cancer patients: preliminary report

**DOI:** 10.1054/bjoc.2001.1747

**Published:** 2001-04

**Authors:** A C Heatherington, J Schuller, A J Mercer

**Affiliations:** Amgen Inc, One Amgen Center Drive, Thousand Oaks, CA, 91320–1799, USA; Krankenanstalt Rudolstiftung, Medizinische Abteilung – Onkologie, Juchgasse 25, Vienna 1030, Austria; Amgen Ltd, Milton Road, Cambridge CB4 OWD, UK

**Keywords:** cancer, chemotherapy, clinical study, darbepoetin alfa, pharmacokinetics

## Abstract

Anaemia is a common occurrence in patients with cancer, and currently can be treated in several ways. Novel erythropoiesis stimulating protein (NESP, darbepoetin alfa) was created using site-directed mutagenesis to have 8 more sialic acid side chains than recombinant human erythropoietin (rHuEPO). The additional sialic acid content has resulted in an approximately 3-fold greater half-life relative to rHuEPO in patients with chronic renal failure. This study evaluates the pharmacokinetic profile of NESP in patients receiving multiple cycles of chemotherapy. Anaemic patients (haemoglobin ≤ 11.0 g dl^−1^) who had non-myeloid malignancies received NESP weekly (2.25 mcg kg^−1^ wk^−1^) under the supervision of a physician, starting on day 1 of chemotherapy for 3 chemotherapy cycles given at 3-week intervals. Blood samples were collected during chemotherapy cycles 1 and 3 for pharmacokinetic analysis. All patients were followed for 4 weeks after treatment. NESP was well tolerated by all patients. After a single dose during chemotherapy cycle 1, pharmacokinetic parameters (mean (SD), *n*) for the first 15 patients were: T _max_ 86.1 (22.8) h (*n* = 14); C _max_ 9.0 (5.1) ng ml^−1^(*n* = 14); t _1/2,z_ 32.6 (11.8) h (*n* = 7); CL/F 3.7 (1.0) ml h^−1^ kg^−1^(*n* = 7). The subjects for whom all parameters could be calculated may represent a sub-group of the entire population. Similar results were obtained in cycle 3. In addition, haemoglobin response data suggests that, in this patient population, dosing less frequently than the 3 times weekly doses used for rHuEPO may be possible while improving anaemia. © 2001 Cance Cancer Research Campaign


*British Journal of Cancer* (2001) **84** (Supplement 1), 11–16
© 2001 Cancer Research Campaign
doi: 10.1054/ bjoc.2001.1747, available online at http://www.idealibrary.com on IDEAL®

### Pharmacokinetics of novel erythropoiesis stimulating protein (NESP) in cancer patients: preliminary report

**AC Heatherington[1], J Schuller[2] and AJ Mercer[3]**

[1]Amgen Inc, One Amgen Center Drive, Thousand Oaks, CA 91320–1799, USA; [2]Krankenanstalt Rudolstiftung, Medizinische Abteilung – Onkologie, Juchgasse 25, 1030 Vienna, Austria; [3]Amgen Ltd, Milton Road, Cambridge CB4 OWD, UK

**Summary** Anaemia is a common occurrence in patients with cancer, and currently can be treated in several ways. Novel erythropoiesis stimulating protein (NESP, darbepoetin alfa) was created using site-directed mutagenesis to have 8 more sialic acid side chains than recombinant human erythropoietin (rHuEPO). The additional sialic acid content has resulted in an approximately 3-fold greater half-life relative to rHuEPO in patients with chronic renal failure. This study evaluates the pharmacokinetic profile of NESP in patients receiving multiple cycles of chemotherapy. Anaemic patients (haemoglobin ≤ 11.0 g $dl^{-1}$) who had non-myeloid malignancies received NESP weekly (2.25 mcg $kg^{-1}$ $wk^{-1}$) under the supervision of a physician, starting on day 1 of chemotherapy for 3 chemotherapy cycles given at 3-week intervals. Blood samples were collected during chemotherapy cycles 1 and 3 for pharmacokinetic analysis. All patients were followed for 4 weeks after treatment. NESP was well tolerated by all patients. After a single dose during chemotherapy cycle 1, pharmacokinetic parameters (mean (SD), *n*) for the first 15 patients were: $T_{max}$ 86.1 (22.8) h ($n = 14$); $C_{max}$ 9.0 (5.1) ng $ml^{-1}$ ($n = 14$); $t_{1/2,z}$ 32.6 (11.8) h ($n = 7$); CL/F 3.7 (1.0) ml $h^{-1}$ $kg^{-1}$ ($n = 7$). The subjects for whom all parameters could be calculated may represent a sub-group of the entire population. Similar results were obtained in cycle 3. In addition, haemoglobin response data suggests that, in this patient population, dosing less frequently than the 3 times weekly doses used for rHuEPO may be possible while improving anaemia. © 2001 Cancer Research Campaign

**Keywords**: cancer; chemotherapy; clinical study; darbepoetin alfa; pharmacokinetics

Erythropoietin (EPO), a sialoglycoprotein that regulates the circulating erythroid mass, is produced in the kidneys in response to hypoxia by a regulated feedback mechanism. Administration of recombinant human erythropoietin (rHuEPO) has been shown to be effective in amelioration of the anaemia associated with renal disease and the anaemia associated with chemotherapy (Platanias et al, 1991; Miller et al, 1992; Cascinu et al, 1994; Henry and Abels, 1994; Ludwig et al, 1995; Del Mastro et al, 1997; Glaspy et al, 1997; Henry, 1998; Maraveya and Pettengell, 1998).

Novel erythropoiesis stimulating protein (NESP, darbepoetin alfa) was developed recently and exhibits extended pharmacokinetic properties compared with rHuEPO. NESP, which has 8 more sialic acids than rHuEPO, and is therefore biochemically distinct, has an increased terminal half-life in animal models and in patients undergoing peritoneal dialysis (Macdougall et al, 1999; Macdougall, 2000). Increasing the half-life of active drug may offer a potential clinical advantage by allowing less frequent dosing.

It was anticipated that the pharmacokinetics of NESP in patients with cancer would be similar to those observed for patients with renal failure. This hypothesis was based on preclinical studies that indicated that the clearance pathway of NESP, like rHuEPO, is primarily by desialylation and elimination through the liver (Spivak and Hogans, 1989). However, patients with cancer have many confounding factors, including the administration of chemotherapy and subsequent myelosuppression, that may have unanticipated effects on pharmacokinetic parameters. In addition, the depletion of responding erythroid precursors by the cytotoxic effects of chemotherapy may result in a higher dosing requirement than is needed in the setting of renal failure. Although the pharmacokinetics of rHuEPO in dialysis patients have been described, no such report exists for the pharmacokinetics of rHuEPO in patients with cancer (Macdougall et al, 1989). The current study was undertaken to evaluate the pharmacokinetic profile of NESP administered to cancer patients receiving multiple cycles of chemotherapy and to compare the pharmacokinetics of single- and multiple-dose administration of NESP through 3 cycles of chemotherapy. This report describes results from the first 15 patients treated.

### MATERIALS AND METHODS

### Patients

Before enrolling patients in the study, independent ethics committee approval and written patient informed consent were obtained. Patients with non-myeloid malignancies were eligible to enrol if they were scheduled to receive cyclic chemotherapy at 3-week intervals for at least 3 more cycles and had a haemoglobin value ≤ 11.0 g $dl^{-1}$, a life expectancy of at least 3 months, and an Eastern Cooperative Oncology Group (ECOG) performance status of 0 to 2. Other eligibility requirements included adequate renal (creatinine ≤ 2.0 mg $dl^{-1}$) and hepatic (bilirubin ≤ 1.5 times upper limit of normal range) function. Patients were to be excluded from

*Correspondence to:* Dr AC Heatherington

This study was supported by Amgen Inc, Thousand Oaks, California.

the study if they had a history of primary haematologic disorders that could cause anaemia; clinically significant disease or dysfunction of the cardiovascular, pulmonary, endocrine, neurological, gastrointestinal or genitourinary systems; primary or metastatic malignancy involving the central nervous system; uncontrolled hypertension; seizures; or iron deficiency.

Twenty to 30 patients were to be enrolled from eight centres. Patients who responded to treatment could elect to continue NESP treatment until completion of their course of chemotherapy (a maximum of 24 weeks). Data from this optional portion of the study concerning haemoglobin response are not presented in this report, but adverse event data are reported from time of enrolment to end of follow-up period.

### Study design

This was an open-label study of NESP (darbepoetin alfa, ARANESP™, Amgen Inc, Thousand Oaks, CA) administered by weekly subcutaneous injection to patients receiving multiple cycles of chemotherapy. This report includes the results of the first 15 patients enrolled, who had completed study drug administration by November 2000. Patients had a medical history and physical examination, including ECOG performance status determination, at screening. Endogenous EPO levels were measured on study day 1 before administration of NESP. Patients received NESP 2.25 mcg $kg^{-1}$ $wk^{-1}$, under the supervision of a physician, as a single subcutaneous injection immediately before receiving chemotherapy, and through 3 cycles of chemotherapy, which were at least 3 weeks apart (Part A). The first dose of NESP was administered on day 1 of the next cycle of chemotherapy after enrolment. Patients who responded to treatment could elect to continue NESP treatment until completion of their course of chemotherapy to a maximum of 24 weeks (Part B).

Blood samples were collected over a 7-day period on week 1 of chemotherapy cycles 1 and 3 to determine the pharmacokinetic profile of NESP. Blood samples for pharmacokinetic analyses were drawn before NESP administration and at 6, 24, 32, 48, 72, 96 and 120 hours after NESP administration. In addition, weekly pre-dose/trough (168 hour) samples and additional 48-hour post-dose (cycle 2) samples were collected. Samples for complete blood count (including reticulocytes) were collected at screening and weekly during the study to assess haemoglobin response. Blood samples for detection of antibody formation were collected before study drug administration on day 1 of cycle 1 (baseline) and on day 1 of cycle 3 before administration of NESP and chemotherapy. Additional samples for the detection of antibody formation were collected throughout the optional treatment phase and at the end of study.

The frequency of red blood cell transfusions was recorded. Adverse events and determinations of serum antibodies to NESP were monitored. All patients had a 4-week follow-up period after the end of study drug administration.

### Dose adjustments

If, during the study, a patient's haemoglobin value increased by ≥ 2.0 g $dl^{-1}$ over any 4-week period in the absence of red blood cell transfusion, the dose of NESP was reduced by 50%. In addition, if the patient's haemoglobin value increased to > 15.0 g $dl^{-1}$ (for men) or > 14.0 g $dl^{-1}$ (for women), the dose of NESP was withheld until the haemoglobin value decreased to ≤ 12.0 g $dl^{-1}$; study drug was then resumed at 50% of the previous dose level.

Patients who did not respond (i.e. had ≤ 1 g $dl^{-1}$ change from baseline) after 6 weeks (2 cycles) were to have the dose of NESP increased to 4.5 mcg $kg^{-1}$ $wk^{-1}$.

### Determination of EPO and NESP serum concentrations

The assays to determine endogenous EPO serum concentrations and NESP serum concentrations were conducted by MDS Pharma Services (Quebec, Canada) using a standard commercial ELISA kit. The antibodies in the Quantikine rHuEPO kit (R & D Systems, Minneapolis, MN) are able to detect both EPO and NESP. The ELISA assay uses a monoclonal antibody immobilized to the plate and a sandwich polyclonal antibody for detection. The colourimetric read-out (optical density) was measured at 450 to 650 nm, wherein the colour change was directly proportional to the amount of endogenous EPO or NESP in the sample. Each sample was run in duplicate with 7 standards and 5 quality-control samples on the plate; these samples were prepared in buffer. Specimen diluent was used to dilute samples as needed.

For endogenous EPO, the standard curve ranged from 10.080 to 380.800 mU $ml^{-1}$, with a lower limit of quantification of 12.000 mU $ml^{-1}$. The assay has been validated, demonstrating accuracy (intra-assay: 98% to 114%; interassay: 98% to 108%, where values are percentage of nominal concentration of quality controls), precision (intra-assay: 4% to 14%; interassay: 2% to 10%, where values are percent coefficient of variation for quality control), and stability (five freeze-thaw cycles). Of the tested substances, (recombinant human [rHu] interleukin-3 [rHuIL-3], rHuIL-6, rHu granulocyte-macrophage colony-stimulating factor [rHuGM-CSF], r-metHuG-CSF, and NESP), only NESP cross-reacted in the assay.

For NESP, the standard curve ranged from 0.125 to 5.000 ng $ml^{-1}$, with a lower limit of quantification of 0.140 ng $ml^{-1}$. The assay has been validated, demonstrating accuracy (intra-assay: 107% to 112%; interassay: 105% to 110%, where values are percentage of nominal concentration of quality controls), precision (intra-assay: 3% to 17%; interassay: 3% to 6%, where values are percent coefficient of variation for quality control), and stability (five freeze-thaw cycles). Of the tested substances (rHuIL-3, rHuIL-6, rHuGM-CSF, r-metHuG-CSF and rHuEPO), only rHuEPO cross-reacted in the assay.

### Pharmacokinetic analysis

All values that were below the lower limit of quantification (generally 0.14 ng $ml^{-1}$ for NESP) were recorded as zero. In all cases, measured NESP serum concentrations were baseline corrected to account for the presence of endogenous EPO, which cross-reacts in the NESP assay. When baseline correction yielded a negative value, this value was recorded as zero. This method of correction assumes that the endogenous EPO concentration remains constant over time for a given individual.

Pharmacokinetic analysis was performed (WinNonLin Pro, Pharsight, Palo Alto, CA) using non-compartmental analysis techniques. The pharmacokinetic parameters determined were maximum observed concentration ($C_{max}$) and the time at which this concentration occurred ($T_{max}$). Relative clearance (CL/F) was determined from ($AUC_{(0-\infty)}$/Dose), and terminal half-life ($t_{1/2,z}$) was estimated by log-linear regression of the terminal portion of the curve.

 © 2001 Cancer Research Campaign

### Statistical analysis

The number and proportion of patients achieving haemoglobin response at the end of 6 weeks (change from baseline of > 1.0 g $dl^{-1}$ in the absence of red blood cell transfusions) and at the end of the third chemotherapy cycle (change from baseline of ≥ 2.0 g $dl^{-1}$ in the absence of red blood cell transfusions) were tabulated. Exact 95% confidence intervals are given for the proportions.

The number and proportion of men reaching a haemoglobin concentration of > 15.0 g $dl^{-1}$ and the number and proportion of women reaching a haemoglobin concentration of > 14.0 g $dl^{-1}$ are presented. Summary statistics (mean, standard deviation (SD), median, quartiles, minimum and maximum) were tabulated for the maximum haemoglobin concentration achieved by the end of the third chemotherapy cycle and the maximum change from baseline over the same period.

The patient incidence of adverse events was calculated by body system and preferred term for events that started or worsened on or after the day of study drug administration and before or at the end of follow-up. Notable results are presented here; the complete tabulation is on file at Amgen Ltd.

### RESULTS

### Patient demographics and disposition

Patient characteristics and demographic information are given in Table 1. Available data from 15 patients are included for evaluation of haemoglobin response, and data from 15 and 7 patients are included for pharmacokinetic analysis for cycle 1 and cycle 3, respectively. 5 patients discontinued study; 2 because of death due to disease progression (reported by the investigator as unrelated to NESP), 1 because of an adverse event of asthenia (reported by the investigator as unrelated to NESP) and 2 for administrative reasons.

**Table 1** Demographics and baseline characteristics for patients receiving NESP 2.25 mcg $kg^{-1}$ $wk^{-1}$ who completed the study by the November 2000 interim analysis

| | NESP (2.25 mcg $kg^{-1}$ $wk^{-1}$) |
|---|---|
| Number of patients enrolled, *n* | 15 |
| Age (years) | |
| Mean (SD) | 61.3 (12.7) |
| Sex, *n* (%) | |
| Women | 8 (53%) |
| Men | 7 (47%) |
| Race | |
| White | 15 (100%) |
| Weight (kg) | |
| Mean (SD) | 67.8 (14.0) |
| Tumour type, *n* (%) | |
| Non-Hodgkin's lymphoma | 3 (20%) |
| Hodgkin's lymphoma | 2 (13%) |
| Lung | 3 (20%) |
| Breast | 2 (13%) |
| Gastrointestinal | 2 (13%) |
| Gynaecologic | 2 (13%) |
| Genitourinary | 1 (6.7%) |
| ECOG performance status | |
| 0 | 6 (40%) |
| 1 | 7 (47%) |
| 2 | 2 (13%) |
| Baseline Hgb (g $dl^{-1}$) | |
| Mean (SD) | 9.6 (0.8) |
| Minimum, maximum | 8.3, 10.5 |

ECOG = Eastern Cooperative Oncology Group; Hgb = haemoglobin; SD = standard deviation.

### Results of pharmacokinetic analysis

Baseline endogenous EPO concentrations were determined using both the EPO and NESP assays. The mean (standard deviation (SD)) endogenous EPO concentrations, assessed against an EPO standard curve, were 67.6 (69.4) mU $ml^{-1}$ ($n$ = 15). The minimum and maximum values were 0.00 and 235 mU $ml^{-1}$, respectively. When assessed using the NESP standard curve, mean (SD) prestudy cross-reactivity in the assay was 0.774 (0.727) ng $ml^{-1}$ ($n$ = 15).

Summary statistics for non-compartmental pharmacokinetic parameters after single doses (week 1, cycle 1) are provided in Table 2. Cycle 1, week 1 intensive profiles were determined for 15 patients, but only 8 of these profiles were evaluable. In 5 of 15 patients, the 168-hour sampling period was insufficient to accurately determine extrapolated parameters (percentage extrapolated > 25%). In 2 additional patients, the terminal phase could not be determined because the serum NESP concentrations did not decrease consistently. Therefore, values for terminal half-life ($t_{1/2, z}$) and relative clearance (CL/F) were estimated for 8 patients. Additionally, 1 of the 8 patients for whom all pharmacokinetic parameters were estimated had extremely low serum NESP concentrations compared with the other patients, resulting in low $C_{max}$ and high CL/F. For this reason, all summary statistics were calculated with and without this patient.

Data are presented as mean (SD). After single-dose (cycle 1) administration (2.25 µg $kg^{-1}$), the mean maximal NESP concentration of 8.44 (5.3) ng $ml^{-1}$ occurred at 85.1 (22.3) hours after NESP administration, the terminal half-life ranged from 20.7 to 54.2 hours (33.1 (11.1) hours), and the relative clearance ranged from 2.37 to 22.4 ml $h^{-1}$ $kg^{-1}$ (6.03 (6.66) ml $h^{-1}$ $kg^{-1}$) (data not shown). Exclusion of the 1 patient with low pharmacokinetic parameters resulted in mean maximal concentration of 8.96 (5.10) ng $ml^{-1}$ that occurred at 86.1 (22.8) hours after NESP administration, a terminal half-life that ranged from 20.7 to 54.2 hours (32.6 (11.8) hours), and a relative clearance that ranged from 2.37 to 5.49 ml $h^{-1}$ $kg^{-1}$ (3.7 (1.0) ml $h^{-1}$ $kg^{-1}$).

Baseline-corrected weekly trough concentrations before the administration of NESP 2.25 mcg $kg^{-1}$ $wk^{-1}$ are provided in Figure 1 for patients receiving chemotherapy every 3 weeks. Within each chemotherapy cycle, the trough concentrations varied, increasing from minimum values at week 1 to maximum values at week 2

**Table 2** Summary statistics for non-compartmental pharmacokinetic parameters, week 1 of cycle 1. Data from one patient with extremely low concentrations have been omitted

| Parameter | $T_{max}$ (hours) | $C_{max}$ (ng $ml^{-1}$) | $t_{1/2, z}$ (hours) | CL/F (ml $h^{-1}$ $kg^{-1}$) |
|---|---|---|---|---|
| Mean | 86.1 | 8.96 | 32.6 | 3.70 |
| SD | 22.8 | 5.10 | 11.8 | 0.995 |
| % CV | 26.5 | 56.9 | 36.4 | 26.9 |
| *n* | 14 | 14 | 7 | 7 |

$T_{max}$ = time to maximum concentration; $C_{max}$ = maximum serum concentration; $t_{1/2, z}$ = terminal half-life; CL/F = relative clearance.

© 2001 Cancer Research Campaign

(approximately 3-fold increase) and then declining on week 3. As expected, a general trend of increasing trough concentrations over time (cycle 3 demonstrating an approximate 2-fold increase over cycle 1) was observed as steady-state concentrations were achieved.

Mean (SD) NESP serum concentration–time profiles for cycle 1 and cycle 3 after administration are compared in Figure 2. For most patients, the serum concentration–time profiles were typical of those seen after subcutaneous dosing: a slow increase to peak concentration (range of single dose: 48 to 123 hours post-dose) and subsequent monophasic decline. Comparison of the mean profiles after single and multiple dosing indicated that NESP pharmacokinetics do not alter substantially after multiple dosing, given the variability in the dataset. However, the higher concentrations at the earlier time points during cycle 3 are a result of increasing concentrations to steady state.

Full non-compartmental analysis was not conducted on the cycle 3 profiles because of inadequate sampling time (168 hours) and the extended elevation of the serum concentrations. However, comparison of mean (SD) maximal concentrations for cycle 1 (8.44 (5.3) ng $ml^{-1}$, $n$ = 15) and cycle 3 (10.3 (8.0) ng $ml^{-1}$, $n$ = 7) indicates a slight increase in concentration, reflective of increase in steady state.

The mean (SD) change of haemoglobin values from baseline was 2.3 (1.2) g $dl^{-1}$ at the end of Part A and 1.2 (1.1) at the end of 4 weeks (Table 3). Haemoglobin values were monitored and the dose of NESP was modified during the study, depending on the haemoglobin response observed. Only 1 patient exceeded pre-determined threshold levels for discontinuation of study drug: a woman with a haemoglobin value > 14 g $dl^{-1}$.

### Safety

The type and incidence of adverse events reported in this study are consistent with the population being studied. No unexpected trends in the type, incidence or severity of adverse events were observed. The most frequently reported adverse events were asthenia (53%), vomiting (47%), nausea (47%) and fever (40%).

Two of the 15 patients (13%) died of disease progression; both deaths were reported by the investigator to be unrelated to NESP administration. One of these patients died after completing 10 weeks of study drug administration, and 1 died on study after week 5 of study drug administration period. Both of these patients are included in the safety analysis.

Six patients reported adverse events meeting the International Conference on Harmonisation (ICH) definition of serious, including neutropenic fever, shortness of breath, congestive heart failure, angina, deep vein thrombosis and erysipelas. None of these serious adverse events were considered by the investigator to be related to NESP. No patients were identified as having developed antibodies to NESP.

### DISCUSSION

This preliminary pharmacokinetic analysis of NESP in patients with non-myeloid malignancies indicates that NESP is slowly absorbed after subcutaneous administration, reaching peak concentrations approximately 85 hours after NESP administration. In the evaluable sub-group, NESP exhibits low relative clearance (mean 3.7 ml $h^{-1}$ $kg^{-1}$) and a long terminal half-life (mean 32.6 hours). The inability to estimate the terminal phase during the 168-hour sampling period for a number of patients biases the

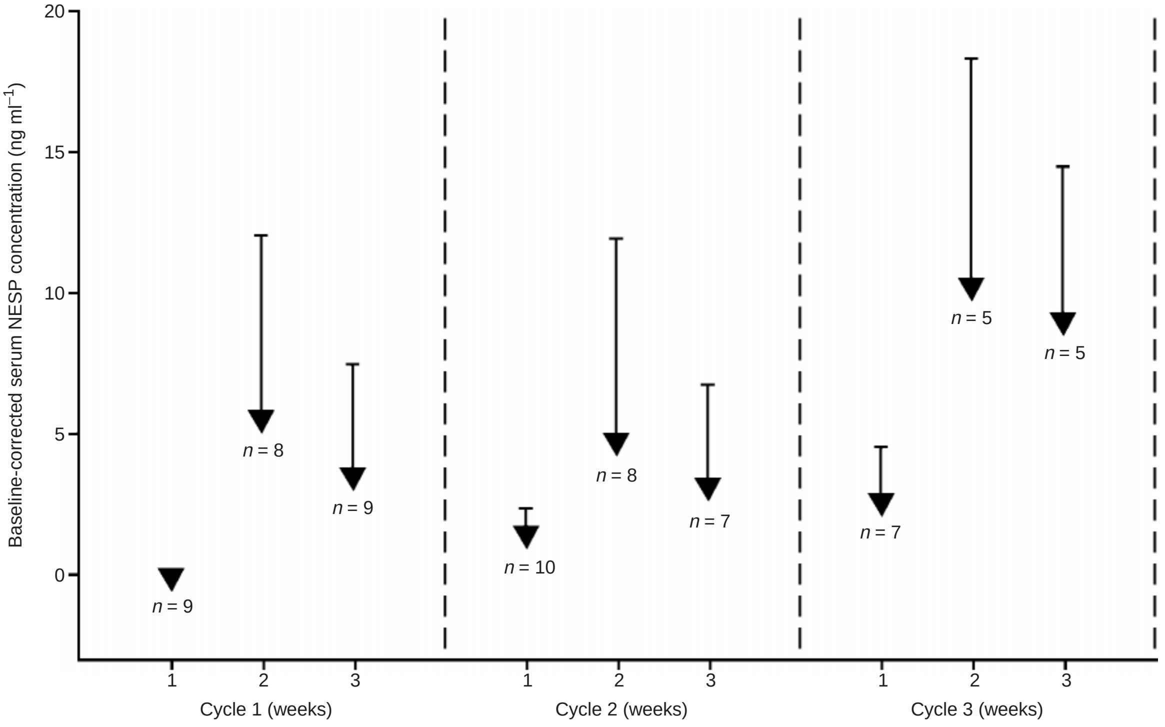

**Figure 1** Mean (standard deviation) baseline-corrected serum NESP trough concentrations (ng $ml^{-1}$) for weekly subcutaneous administration of 2.25 mcg $kg^{-1}$ $wk^{-1}$ NESP for patients with 3-week chemotherapy cycles. Error bars are one standard deviation

*© 2001 Cancer Research Campaign*

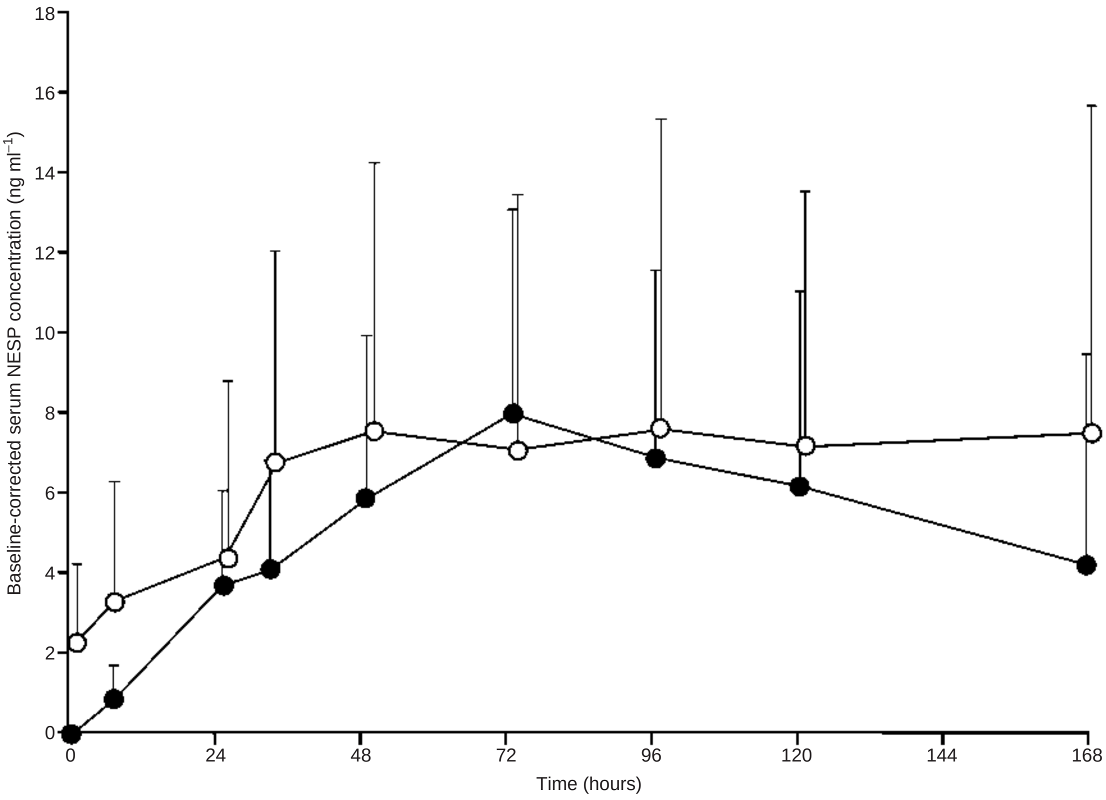

**Figure 2** Mean (standard deviation) baseline-corrected serum NESP concentration (ng ml$^{-1}$) after weekly subcutaneous administration of 2.25 mcg kg$^{-1}$ wk$^{-1}$ NESP, week 1 of cycle 1 and week 1 of cycle 3. Solid circles, cycle 1, *n* = 14 to 15; open circles, cycle 3, *n* = 7 to 9. Error bars are one standard deviation actual estimated values toward those patients with a shorter terminal half-life. Hence, the subjects for whom the NESP serum concentration–time profiles were evaluable over the 168-hour interval (8 of 15) may represent a sub-group of the entire population. A longer sampling duration and dosing interval would be required to accurately estimate the half-life in patients with cancer receiving chemotherapy in whom NESP exhibits a longer terminal half-life, i.e. a second sub-group whose clearance and/or absorption is very slow. Overall, increases in serum concentrations reflect increases to steady-state values, as verified by pharmacokinetic modelling. These results suggest that it may be possible to administer NESP at less frequent intervals and still maintain the observed biologic effect. Studies are ongoing to assess the efficacy of NESP at less frequent dose schedules.

**Table 3** Haemoglobin response

| | NESP (2.25 mcg kg$^{-1}$ wk$^{-1}$) |
|---|---|
| Number of patients enrolled, *n* | 15 |
| Mean (SD) change in Hgb by week 4 g dl$^{-1}$ | 1.2 (1.1) |
| Mean (SD) change in Hgb by end of study Part A g dl$^{-1}$ | 2.3 (1.2) |
| Total number of women, *n* | 8 |
| Number with Hgb > 14 g dl$^{-1}$ | 1 (13%) |
| Total number of men, *n* | 7 |
| Number with Hgb > 15 g dl$^{-1}$ | 0 (0%) |

Hgb = haemoglobin; SD = standard deviation.

The pharmacokinetic properties of NESP, with both intravenous and subcutaneous dosing, have previously been determined in patients with chronic renal failure (Macdougall et al, 1999). After subcutaneous administration of a single dose of NESP 0.5 mcg kg$^{-1}$, mean (SD) peak serum concentrations of 0.94 (0.1) ng ml$^{-1}$ were observed at approximately 50 hours after dosing. The terminal half-life was 48.8 (5.2) hours, and relative clearance (calculated using mean intravenous clearance and mean subcutaneous bioavailability) was 4.3 ml h$^{-1}$ kg$^{-1}$. The pharmacokinetics of NESP in oncology patients have been studied in a small number of patients in a dose-ranging study (Glaspy et al, 2000). The terminal half-life of NESP (in 5 patients after a single subcutaneous dose of 0.5, 1.5 or 4.5 mcg kg$^{-1}$ NESP) ranged from 30.1 to 45.6 hours, and the relative clearance ranged from 1.86 to 4.01 ml h$^{-1}$ kg$^{-1}$. These limited data span the values obtained in the current study and those obtained in studies in the chronic renal failure setting.

The differences in terminal half-life between the patient population with chronic renal failure and the current study most likely reflect the bias towards lower estimates in the current study, especially since the relative clearance values are similar. However, the possibility that the pharmacokinetics of NESP differ somewhat between patients with chronic renal failure and patients with cancer receiving chemotherapy cannot be excluded. These differences could result from the disease state itself or from the concomitant administration of chemotherapy. The observation in the current study that NESP trough serum concentrations varied within a chemotherapy cycle indicated the possible impact of

*© 2001 Cancer Research Campaign*

myelosuppression on the pharmacokinetics of NESP. Within a cycle, the week 1 values (immediately before both NESP and chemotherapy administration) were the lowest of any cycle, whereas the week 2 values (1 week after chemotherapy) were the highest of any cycle. These observations point to the possibility of bone marrow involvement in either the clearance or distribution of NESP, perhaps through the EPO receptor on the erythroid precursors. Immediately after chemotherapy, the pool of erythroid precursors decreases, allowing more NESP to be circulating and thus measurable. Additional preclinical studies are ongoing to elucidate these mechanisms, as well as clinical studies in patients with cancer not receiving chemotherapy. The possibility of bone marrow involvement in the catabolism of endogenous EPO (Piroso et al, 1989) and an observed increase in endogenous EPO due to myeloablation (Piroso et al, 1989; Sawabe et al, 1996) have been previously published.

The haemoglobin response of patients receiving NESP in this study suggests that a dose of 2.25 mcg $kg^{-1}$ $wk^{-1}$ is an effective initial dose in this population and that modification of the dosing schedule, depending upon the haemoglobin value, allowed an appropriate response to be maintained over the course of the chemotherapy administered. The mean changes in haemoglobin observed at week 4 and at the end of Part A of the study compare favourably with data cited in the literature for rHuEPO as well as with data presented in another paper in this supplement (Glaspy et al, 2001).

The available safety data from this study suggest that NESP 2.25 mcg $kg^{-1}$ $wk^{-1}$ is well tolerated in this population. The adverse events reported in this study are typical for this patient population with advanced malignancy receiving multicycle chemotherapy, and their pattern, incidence and severity are similar to those labelled for rHuEPO. No antibodies to NESP were detected.

This study continues to enrol patients to further explore the pharmacokinetics of NESP after both subcutaneous and intravenous administration. Based on these preliminary findings, the pharmacokinetic parameters of NESP suggest that it has a low clearance and a prolonged half-life that may allow for infrequent administration of this agent to patients with cancer who are receiving multicycle chemotherapy.

### ACKNOWLEDGEMENTS

Robert Rovetti, BS, assisted with the pharmacokinetic analysis; Jose Rodriguez, BS, was responsible for the oversight of the NESP ELISA; and John Ferbas, PhD, was responsible for the antibody analysis. Peter Treasure assisted with the statistical analysis. Alan B Colowick, MD, MPH; Janet L Nichol, MS; and MaryAnn Foote, PhD, assisted with the writing of this manuscript.

### REFERENCES

Cascinu S, Fedeli A, Del Ferro E, Luzi Fedeli S and Catalano G (1994) Recombinant human erythropoietin treatment in cisplatin-associated anemia: a randomized double-blind trial with placebo. *J Clin Oncol* **12**: 1058–1062

Del Mastro L, Venturini M, Lionetto R, Garrone O, Melioli G, Pasquetti W, Sertoli MR, Bertelli G, Canavese G, Costantini M and Rosso R (1997) Randomized phase III trial evaluating the role of erythropoietin in the prevention of chemotherapy-induced anemia. *J Clin Oncol* **15**: 2715–2721

Glaspy J, Bukowski R, Steinberg D, Taylor C, Tchekmedyian S and Vadhan RS (1997) Impact of therapy with epoetin alfa on clinical outcomes in patients with nonmyeloid malignancies during cancer chemotherapy in community oncology practice. *J Clin Oncol* **15**: 1218–1234

Glaspy J, Colowick AB and Heatherington A (2000) Novel erythropoiesis stimulating protein (NESP) exhibits a prolonged serum half-life ($T_{1/2}$) in oncology patients (pts). *Proc Am Soc Clin Oncol* **19**: 54a (abstract 210)

Glaspy J, Jadeja JS, Justice G, Kessler J, Richards D, Schwartzberg L, Rigas J, Kuter D, Harmon D, Prow D, Demetri G, Gordon D, Arseneau J, Saven A, Hynes H, Boccia R, O'Byrne J and Colowick AB (2001) A dose-finding and safety study of novel erythropoiesis stimulating protein (NESP) for the treatment of anaemia in patients receiving multicycle chemotherapy. *Br J Cancer* **84** (Supp 1): 17–23

Henry DH (1998) Epoetin alfa and high-dose chemotherapy. *Semin Oncol* **25**: 54–57

Henry DH and Abels RI (1994) Recombinant human erythropoietin in the treatment of cancer and chemotherapy-induced anemia: Results of double-blind and open-label follow-up studies. *Semin Oncol* **21**: 21–28

Ludwig H, Sundal E, Pecherstorfer M, Leitgeb C, Bauernhofer T, Beinhauer A, Samonigg H, Kappeler AW and Fritz E (1995) Recombinant human erythropoietin for the correction of cancer associated anemia with and without concomitant cytotoxic chemotherapy. *Cancer* **76**: 2319–2329

Macdougall IC (2000) Novel erythropoiesis stimulating protein. *Semin Nephrol* **20**: 375–381

Macdougall IC, Roberts DE, Neubert P, Dharmasena AD, Coles GA and Williams JD (1989) Pharmacokinetics of recombinant human erythropoietin in patients on continuous ambulatory peritoneal dialysis. *Lancet* **1**: 425–427

Macdougall IC, Gray S, Elston O, Breen C, Jenkins B, Browne J and Egrie J (1999) Pharmacokinetics of novel erythropoiesis stimulating protein compared with Epoetin alfa in dialysis patients. *J Am Soc Nephrol* **10**: 2392–2395

Maraveya S and Pettengell R (1998) What is the role of erythropoietin in patients with solid tumours? *Ann Oncol* **9**: 239–241

Miller CB, Platanias LC, Mills SR, Zahurak ML, Ratain MJ, Ettinger DS and Jones RJ (1992) Phase I–II trial of erythropoietin in the treatment of cisplatin-associated anemia. *J Natl Cancer Inst* **84**: 98–103

Piroso E, Erslev AJ and Caro J (1989) Inappropriate increase in erythropoietin titers during chemotherapy. *Am J Hematol* **32**: 248–254

Platanias LC, Miller CB, Mick R, Hart RD, Ozer H, McEvilly JM, Jones RJ and Ratain MJ (1991) Treatment of chemotherapy-induced anemia with recombinant human erythropoietin in cancer patients. *J Clin Oncol* **9**: 2021–2026

Sawabe Y, Kikuno K, Iseki T, Iida S, Tabata Y and Yonemitsu H (1996) Changes in serum erythropoietin and the reticulocyte count during chemotherapy for leukemias. *Eur J Haematol* **57**: 384–388

Spivak JL and Hogans BB (1989) The in vivo metabolism of recombinant human erythropoeitin in the rat. *Blood* **73**: 90–99

 © 2001 Cancer Research Campaign
